# *Pantoeaagglomerans*-Infecting Bacteriophage vB_PagS_AAS21: A Cold-Adapted Virus Representing a Novel Genus within the Family *Siphoviridae*

**DOI:** 10.3390/v12040479

**Published:** 2020-04-23

**Authors:** Monika Šimoliūnienė, Lidija Truncaitė, Emilija Petrauskaitė, Aurelija Zajančkauskaitė, Rolandas Meškys, Martynas Skapas, Algirdas Kaupinis, Mindaugas Valius, Eugenijus Šimoliūnas

**Affiliations:** 1 Department of Molecular Microbiology and Biotechnology, Institute of Biochemistry, Life Sciences Center, Vilnius University, Saulėtekio av. 7, LT-10257 Vilnius, Lithuania; monika.simoliuniene@gmc.vu.lt (M.Š.); petrauskaitemilija@gmail.com (E.P.); aurelija.zajanckauskaite@bchi.vu.lt (A.Z.); rolandas.meskys@bchi.vu.lt (R.M.); 2 Center for Physical Sciences and Technology, Saulėtekio av. 3, LT-10257 Vilnius, Lithuania; martynas.skapas@gmail.com; 3 Proteomics Centre, Institute of Biochemistry, Life Sciences Center, Vilnius University, Saulėtekio av. 7, LT-10257 Vilnius, Lithuania; algirdas.kaupinis@gf.vu.lt (A.K.); mindaugas.valius@bchi.vu.lt (M.V.)

**Keywords:** *Pantoea agglomerans*, vB_PagS_AAS21, low temperature bacteriophage, *Siphoviridae*

## Abstract

A novel cold-adapted siphovirus, vB_PagS_AAS21 (AAS21), was isolated in Lithuania using *Pantoea agglomerans* as the host for phage propagation. AAS21 has an isometric head (~85 nm in diameter) and a non-contractile flexible tail (~174 × 10 nm). With a genome size of 116,649 bp, bacteriophage AAS21 is the largest *Pantoea*-infecting siphovirus sequenced to date. The genome of AAS21 has a G+C content of 39.0% and contains 213 putative protein-encoding genes and 29 genes for tRNAs. A comparative sequence analysis revealed that 89 AAS21 open reading frames (ORFs) code for unique proteins that have no reliable identity to database entries. In total, 63 AAS21 ORFs were functionally annotated, including those coding for the proteins responsible for virion morphogenesis, phage-host interactions, and DNA metabolism. Proteomic analysis led to the experimental identification of 19 virion proteins, including 11 that were predicted by bioinformatics approaches. Based on comparative phylogenetic analysis, AAS21 cannot be assigned to any genus currently recognized by ICTV and may represents a new branch of viruses within the family *Siphoviridae*.

## 1. Introduction

Plants are usually populated by complex microbial communities composed of algae, fungi, bacteria, and their viruses. The genus *Pantoea* is one of the predominant taxa in plant microbiomes and comprises both pathogenic and beneficial species that can survive as epiphytes or endophytes on their hosts, but sometimes can cause infections in humans [1,2]. Therefore, bacteriophages could be used as tools for prevention and therapy in both plants and humans or for food preservation [3,4]. Despite their promising potential, only a limited number of *Pantoea*-infecting bacteriophages with completely sequenced genomes, annotated as *Pantoea, Erwinia* or *Serratia* phages, have been published [5,6,7,8] or deposited in the public databases (Supplementary Table S1) to date.

In this study, we present biological characteristics and a complete genome analysis of *Pantoea agglomerans*-infecting bacteriophage vB_PagS_AAS21 (below referred by its shorter name AAS21). Bacteriophage AAS21 shows a low-temperature plating profile, with an optimum temperature for plating of about 22 °C, and an ability to form plaques even at 4 °C. Phylogenetic analysis indicates that AAS21 has no close relatives within the family *Siphoviridae*. Thus, the data presented here not only provide the information on *Pantoea* phages, but also may give new insights that deepen our understanding of phage genetics and virus-host interactions in dynamic ecosystems, such as phyllospheres of various plants.

## 2. Materials and Methods

Phage isolation and propagation were performed by using the enrichment of phages in the source material technique and soft agar overlay method as described previously [6]. The adsorption tests were carried out as described by Kropinski et al. [9]. The bacterial strains used in this study for host range determination are listed in Supplementary Table S2. The high-titer phage lysates were purified using a CsCl step gradient as described previously [10]. Purified phage particles were used for morphological analysis of phage virions and DNA extraction. For TEM analysis, AAS21 virions were applied on the carbon-coated nickel grid (Agar Scientific, Essex, UK), stained with two successive drops of 2% uranyl acetate (pH 4.5), dried, and examined in Tecnai G2 F20 X-TWIN transmission electron microscope (FEI, Hillsboro, OR, USA). The AAS21 genomic DNA was isolated from purified phage particles by phenol/chloroform extraction and ethanol precipitation method, as described by Carlson and Miller [11]. The isolated phage DNA was subsequently used in restriction analysis, for PCR, or it was subjected to genome sequencing. The restriction digestion was performed with Bsu15I, Csp6I, DraI, EcoRII, Eco32I, HhaI, MboI, and NdeI restriction endonucleases (Thermo Fisher Scientific, Vilnius, Lithuania), according to the supplier’s recommendations. Analysis of structural proteins of AAS21 was performed using a modified filter-aided sample preparation (FASP) protocol followed by LC-MS/MS analysis, as described previously [12].

The complete genome sequence of AAS21 was determined using Illumina DNA sequencing technology at GenXPro (Frankfurt, Germany). DNA was fragmented using an ultrasound shearing device (Diagenode, Seraing, Belgium). The library was prepared using the TrueQuant DNA library prep kit (GenXPro, Frankfurt, Germany) for end repair and adapter ligation. After purification and size selection of adapter dimers and fragments below 300 bps, the DNA was sequenced on an Illumnia NextSeq500 machine (Illumina, San Diego, CA, USA) with 1 × 75 bps, at least 1.5 M reads were sequenced per library. For filtering and trimming low-quality reads cutadapt [13] was used. The genome assembly was performed using SPAdes Assembler [14]. The reads were assembled into a single linear contig of 116,776 bp with an average coverage of 240. The ends of the contig were confirmed using PCR, followed by Sanger sequencing reactions. A PCR fragment was obtained by the amplification of the AAS21 wild-type genomic DNA using AAS21_F 5’-GCGATCCATATCATGCAAGATC-3’ and AAS21_R 5’-GTTCTATGACCTGCTTTGTAAG-3’ primers. No defined genomic termini were identified, and to preserve gene contiguity, the genome start point was selected of the predicted terminase small subunit gene. The analysis of the genome sequence was performed using the Fasta-Nucleotide, Fasta-Protein, BLASTP, Transeq (http://www.ebi.ac.uk/Tools/st/emboss_transeq), and Clustal Omega (http://www.ebi.ac.uk/Tools/msa/clustalo), as well as HHPred, HHblits, HMMER and Hhsenser, [15,16]). Also, tRNAscan-SE 1.21 (http://lowelab.ucsc.edu/tRNAscan-SE) and ARAGORN (http://130.235.244.92/ARAGORN) were used to search for tRNAs. The codon usage was calculated using Codon Usage Calculator (https://www.biologicscorp.com/tools/CodonUsageCalculator). The open reading frames (ORFs) were predicted with Glimmer v3.02 (https://www.ncbi.nlm.nih.gov/genomes/MICROBES/glimmer_3.cgi) and Geneious Prime 2019 (http://www.geneious.com). Phylogenetic analysis was conducted using MEGA version 7 [17]. The overall nucleotide sequence identity was calculated using PASC [18], and comparative whole-genome sequence alignment was performed using Geneious Prime 2019 Tree Builder and Progressive Mauve [19]. The genome sequence of AAS21 was deposited in GenBank under the accession number MK770119. 

## 3. Results and Discussion

Phage AAS21 was originally isolated from the outwash of jostaberries collected in Lithuania in 2017. *Pantoea agglomerans* strain AUR was used as the host for phage propagation and phage growth experiments. Out of 23 bacterial strains tested to explore the host range of the phage, only *Pantoea agglomerans* bacteria including the type strain 3493 and isolates AUR, BSL, and SER were shown to be sensitive to AAS21 (Supplementary Table S2). The efficiency of plating (e.o.p.) test revealed that AAS21 forms clear plaques about 2.3 mm in diameter at 4–32 °C, and has an optimum temperature for plating of about 22 °C (Supplementary Figure S1). Therefore, based on the characteristics of physiological types of bacteriophages recognized by Seeley and Primrose [20], phage AAS21 belongs to the group of low temperature phages, which are active at or below 30 °C. However, the molecular mechanisms underlying the cold-adaptation of bacterial viruses remain poorly understood, and only a limited number of Enterobacteria phages capable to replicate at lower temperatures have been described to date [6,21,22,23]. Notably, attempts to obtain a one-step growth curve of AAS21 were unsuccessful because of the slow phage adsorption kinetics. Under investigated conditions, only about 30% of the input virions adsorbed during first 5 min, and, after 15 min, as many as 55% of the input virions remained unattached (Supplementary Figure S2).

**Figure 1:**
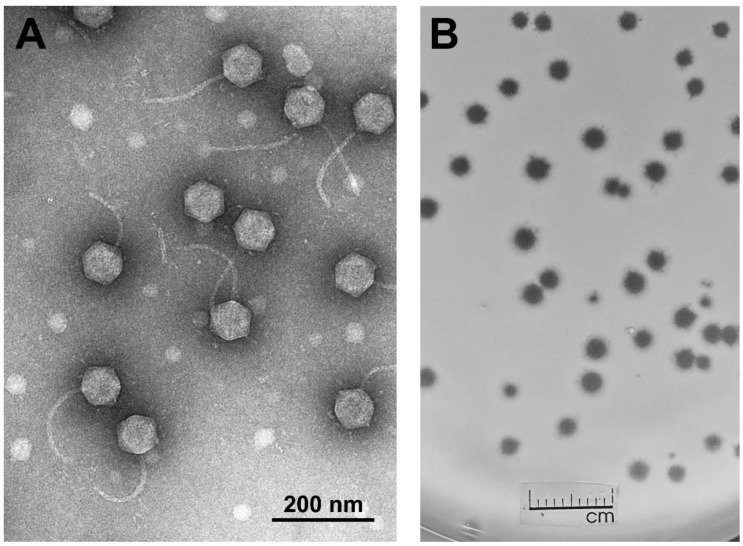
Transmission electron micrograph of AAS21 virions (**A**), and AAS21 plaque morphology (**B**). Plaques formed by AAS21 on a lawn of *Pantoea agglomerans* strain AUR after 24 h of incubation at 22 °C.

TEM analysis revealed that phage AAS21 belongs to the family *Siphoviridae* and is characterized by an isometric head (diameter, 84.75 ± 3.20 nm, [*n* = 50]) and an apparently non-contractile, flexible tail (173.59 ± 11.52 nm in length and 10.27 ± 0.87 nm in width [*n* = 30]) (Figure 1). Although neither the baseplate nor the tail fibers were clearly visible by TEM, several ORFs coding for putative tail fiber proteins have been identified during bioinformatics analysis and/or by proteomics methods (Supplementary Tables S3 and S4).

**Figure 2:**
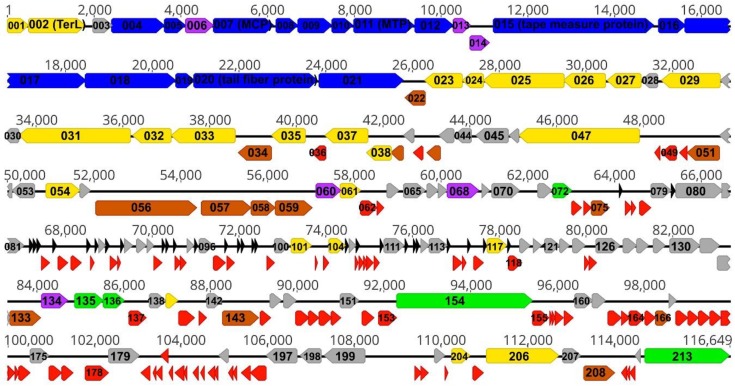
Functional genome map of bacteriophage AAS21. The coding capacity of the AAS21 genome is shown. The numbers indicate gene position in the genome, gene functions are assigned according to the characterized open reading frames (ORFs) in the NCBI database and HHpred analysis. The color code is as follows: yellow—DNA replication, recombination, repair, and packaging; brown—transcription, translation, and nucleotide metabolism; blue—structural proteins; purple—virion morphogenesis-related proteins; green—lysis, phage-host interaction; grey—ORFs of unknown function; red—AAS21 specific ORFs that encode unique proteins with no reliable identity to database entries, black—tRNA.

Bacteriophage AAS21 has a linear, double stranded DNA genome consisting of 116,649 bp with a G+C content of 39.0%, which differs significantly from that (52–55%) observed for *Pantoea* spp. [1]. Among four known *Pantoea* siphoviruses (Supplementary Table S1), the bacteriophage AAS21 becomes the largest one sequenced to date indicating that the diversity of these bacteria-infecting viruses is just starting to be explored. The genome of AAS21 is close-packed – with an average ORF size of 497 bp, 91.7% of the genome is coding. The analysis of the genome sequence revealed that AAS21 has 213 putative protein-encoding genes and 29 genes for tRNA (Figure 2). Notably, there is a certain asymmetry in the distribution of the protein-encoding genes on the two AAS21 DNA strands. In total, 158 AAS21 open reading frames have been predicted to be transcribed from the same DNA strand, whereas 57 ORFs have been found on the opposite strand (Figure 2). A bioinformatics analysis revealed that 89 out of 213 AAS21 ORFs encode unique proteins that have no reliable identity (E-values > 0.001) to the database entries. In the case of AAS21 ORFs that encode proteins with matches to those in other sequenced genomes, the percentage of amino acid identity ranged from 25% to 88% and, in most cases (104 out of 124 AAS21 ORFs), from 38% to 65% (Supplementary Table S3).

Among the AAS21 gene products with detectable homologs in other sequenced genomes, the vast majority (105 out of 124) are most similar to proteins from phages that infect *Salmonella*, *Escherichia*, *Klebsiella*, *Pectobacterium*, *Yersinia,* and *Serratia*. Interestingly, only three AAS21 genes products have the best match with proteins found in bacteriophages infecting *Pantoea* or *Erwinia*. Hence, tail fiber proteins encoded by ORF021, ORF154, and hypothetical protein (ORF176) share the highest identity with a minor tail protein (AVO22985.1) from *Erwinia* phage vB_EamM-Bue1, a putative EPS-depolymerase (QEQ94917.1) from *Erwinia* phage pEp_SNUABM_01 and a hypothetical protein (AZS06388.1) from *Pantoea* phage vB_PagS_AAS23, respectively (Supplementary Table S3). Although a hypothetical protein encoded by ORF080 shares the highest aa identity (57%) with the hypothetical protein (ATS94045.1) from *Pectobacterium* phage DU_PP_V, five out of six AAS21 gp080 best matches (52–51% aa identity) are proteins from *Erwinia* bacteriophages. With an exception of gp176, three out of four aforementioned AAS21 proteins have been identified by LC-MS/MS (Supplementary Table S4) indicating that gp021, gp080, and gp154 might be structural AAS21 proteins, which play an important role in phage–host interactions and could be important factors determining the host range of AAS21.

Based on homology to biologically defined proteins, 63 ORFs of AAS21 were given a putative functional annotation (Supplementary Table S3). A bioinformatics analysis allowed for the identification of 21 genes coding for head (ORF004, ORF007), tail (ORF009–ORF019), tail fibers (ORF020, ORF21, ORF154), and other morphogenesis-associated structural proteins. FASP followed by LC-MS/MS confirmed that all of the predicted structural proteins, except for gp08 (putative head-tail connector), gp009 (putative tail completion protein), gp010 (tail terminator protein), gp12 (putative minor tail protein), gp016 (distal tail protein), and gp017 (putative tail protein) are present in the virion of AAS21 (Supplementary Table S4). The failure to identify aforementioned proteins by proteomics approaches may be due to the incompatibility of these proteins with sample preparation procedures and/or because of their low abundance in virions. On the other hand, the proteomic analysis led to the experimental identification of a number of hypothetical proteins (gp070, gp080, gp081, and gp153) as well as unique proteins of AAS21 with no reliable identity to database entries (gp147, gp172, and gp177) as structural components. In addition, a holin encoded by ORF135 was also identified, suggesting that it might be a virion-associated enzyme.

The bioinformatic analysis revealed that the genes associated with AAS21 DNA packaging, replication, recombination, and repair included those coding for terminase small and large subunits (ORF001 and ORF002, respectively), DNA polymerase I (ORF031), DNA primase (ORF032), DNA ligase subunits A and B (ORF037 and ORF035, respectively), putative ssDNA binding (ORF027), DNA processing (ORF038), PhoH-like (ORF054), A1 (ORF206) and A2 (ORF204) proteins and thioredoxin (ORF139). In addition, two DNA helicases (ORF029 and ORF033), RNA helicase (ORF047), and six nucleases encoded by ORF023, ORF025, ORF026, ORF061, ORF101, and ORF104 have been detected. Phage-host interaction and lysis proteins, identified in the genome of AAS21, included cell wall hydrolase, holin, lysozyme, tail fiber protein–EPS-depolymerase, and receptor binding protein encoded by ORF072, ORF135, ORF136, ORF154, and ORF213, respectively. In addition, a number of genes encoding enzymes involved in the transcription, translation, and nucleotide metabolism have been also detected (Supplementary Table S3). Notably, none of the predicted AAS21 proteins showed sequence homology with integration-related proteins, antibiotic resistance determinants, or virulence factors, indicating that this phage has a potential to be used as a safe biocontrol agent.

In the genome of AAS21, 29 genes coding for tRNA (Supplementary Table S5) together with protein-encoding genes compose a cluster of genes, which is about 14 kb in length (Figure 2). As it was observed in many lytic viruses, tRNA genes usually form clusters, especially in genomes harboring more than 15 tRNAs [24]. The vast majority (30 out of 32) of protein-encoding genes present in this cluster are genes coding for hypothetical (11) or unique (19) proteins with no reliable identity to database entries. In addition, endonuclease V and two HNH endonucleases, encoded by ORF101, ORF104, and ORF117, respectively, are present within the aforementioned cluster of genes. The HNH endonucleases belong to the family of the homing endonuclease that act as a mobile element, and, thus, are supposed to play a role of dissemination of tRNA gene clusters among related organisms [24,25]. The presence of tRNA genes in virus genomes could be associated with numerous functions. Various studies indicated that tRNA genes in viral genomes could be used to compensate for differences in codon and/or amino acid usage between virus and its hosts, resulting in an efficient protein synthesis and/or expansion of the host range [24,25,26,27]. Thus, keeping in mind a significantly lower G+C content (39.0%) of the bacteriophage AAS21 DNA compared to that (52–55%) observed for *Pantoea* spp., it might be speculated that a large number of tRNAs present in the genome of AAS21 are necessary to optimize a translation process. Hence, the genome of AAS21 encodes tRNA^Ser^ with the CGA (based on the results of tRNAscan-SE) or AGA (based on the results of ARAGORN) anticodon. According to the Genomic tRNA Database (GtRNAdb) [28], none of the *Pantoea* spp. genomes analyzed harbors such types of tRNA. Remarkably, the genes of AAS21 encoding the functionally important proteins contain TCG or TCT codons for serine. For example, 0.69, 0.31, and 0.32% of codons in gp072 (cell wall hydrolase), gp021 (tail fiber protein), and gp015 (tape measure protein) encoding genes, respectively, are TCG, while 2.73, 2.45, and 1.94% of codons in gp002 (terminase large subunit), gp031 (DNA polymerase), and gp007 (major capsid protein) encoding genes, respectively, are TCT.

**Figure 3:**
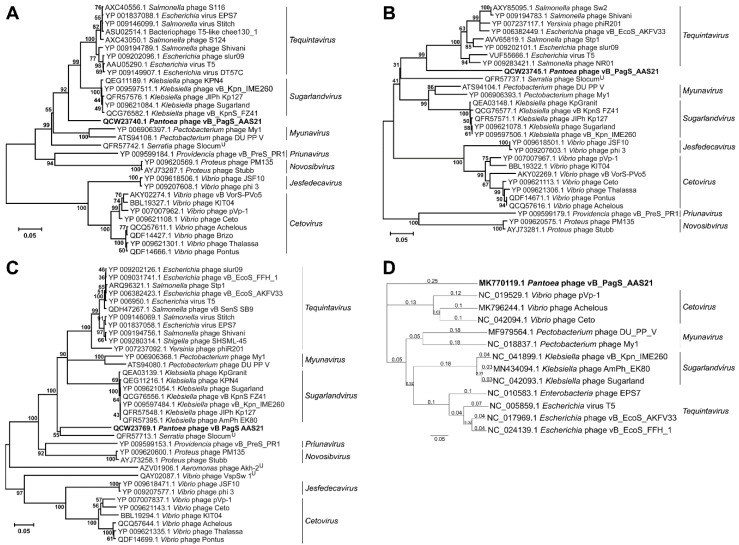
Neighbor-joining tree analysis based on the alignment of the amino acid sequences of: (**A**) Terminase large subunit, (**B**) major capsid protein, and (**C**) DNA polymerase. Phylogenetic analysis was conducted using MEGA version 7. The percentage of replicate trees in which the associated taxa clustered together in the bootstrap test is shown next to the branches. U—unclassified siphoviruses. (**D**) Neighbor-joining tree based on the alignment of AAS21 as well as *Tequintavirus*, *Sugarlandvirus*, *Myunavirus*, and *Cetovirus* phage genome sequences available in NCBI GenBank. The tree was constructed using Geneious Prime 2019 Tree Builder, the numbers at the nodes indicate the bootstrap probabilities.

In order to determine the phylogenetic relationship between AAS21 and its closest relatives, the comparison of the individual genes most often used for the analysis of the evolutionary relationships between bacteriophages [29] was carried out. Therefore, three phylogenetic trees based on the alignment of the AAS21 terminase large subunit, major capsid protein, and DNA polymerase aa sequences with those returned by BLASTP homology searches were constructed (Figure 3). As seen in Figure 3, in all three of the phylogenetic trees, bacteriophage AAS21 represents a distinct branch that seems to occupy a somewhat intermediate position between siphophages belonging to the genera *Tequintavirus*, *Sugarlandvirus*, *Myunavirus*, *Novosibvirus,* and *Priunavirus*. To obtain a more detailed picture of the phylogenetic relationships of AAS21 and its closest relatives, the overall nucleotide sequence identity was calculated using PASC, and a comparative whole-genome sequence alignment was performed using Geneious Prime 2019 Tree Builder and Progressive Mauve. Results of the comparative analysis revealed that the genomes of bacteriophages share several regions of nucleotide sequence similarity that, in AAS21, cover the essential structural and virion morphogenesis proteins-encoding genes, as well as genes related to DNA metabolism and modification (Supplementary Figure S3). Nevertheless, the overall nucleotide sequence similarity between AAS21 and its closest relatives is quite low, and ranges from 41.16% (AAS21 vs. *Escherichia* virus AKFV33) to 36.65% (AAS21 vs. *Salmonella* phage Shivani) (Supplementary Table S6). In addition, AAS21 represents a distinct branch on the neighbor-joining tree, based on the whole-genome sequence alignment of AAS21 and 12 phage genomes analyzed (Figure 3D).

According to the Bacterial and Archaeal Viruses Subcommittee (BAVS) of the ICTV, a genus is described as a cohesive group of viruses sharing a high degree (>50%) of nucleotide sequence similarity [30]. Following this, and, based on the results of the comparative genome sequence analysis performed during this study, bacteriophage AAS21 cannot be assigned to any genus currently recognized by ICTV and likely represents a new one within the family *Siphoviridae*.

In conclusion, we have demonstrated that, to our knowledge, with a genome size of 116,649 bp, AAS21 is the largest *Pantoea*-infecting siphovirus sequenced to date. Also, we have shown that AAS21 is a cold-adapted bacteriophage having no close phylogenetic relationships with other viruses, containing a high number of tRNA and active on number of bacteria from *Pantoea* genus. Thus, these findings do not only provide new data for analyzing phage evolution, but also represent a bacteriophage, which could be a suitable model for studies of the role of phage-encoded tRNAs in translational capacity during infection or still poorly understood molecular mechanisms underlying the cold-adaptation of bacterial viruses.

## Supplementary Materials

The following are available online at https://www.mdpi.com/1999-4915/12/4/479/s1. Table S1: A list of Pantoea bacteriophages with completely sequenced genomes, which have been published and/or deposited in the public databases. Table S2: Bacterial strains used in this study to determine the host range of phage AAS21; Table S3: AAS21 ORFs with homologues in other viruses or cellular organisms. Table S4: AAS21 virion proteins identified by MS. Table S5: AAS21 tRNAs identified with tRNAscan-SE and ARAGORN. Table S6: Top matches for BLAST-based alignments of whole genome sequences of AAS21 and its closest relatives generated using PASC. Figure S1: The effect of temperature on the efficiency of plating of phage AAS21 on the culture of P. agglomerans strain AUR. Figure S2: Adsorption assay of phage AAS21. Figure S3: Progressive Mauve whole-genome alignment generated using Geneious Prime 2019.
