# Supplementary material for: Pantoeaagglomerans-Infecting Bacteriophage vB_PagS_AAS21: A Cold-Adapted Virus Representing a Novel Genus within the Family Siphoviridae
Source: Viruses. 2020 Apr 23;12(4):479. doi: 10.3390/v12040479 (PMC7232348; doi:10.3390/v12040479)
Supplement: Supplementary file 1 [file viruses-12-00479-s001.pdf]

Supplementary Table S1. A list of *Pantoea* bacteriophages with completely sequenced genomes, which have been published and/or deposited in the public databases.

Phage	GenBank accession no.	Family	Genome size (bp)	Reference
vB_PagM_LIET2	MK388689.1	*Myoviridae*	74710	unpublished
vB_PagM_SSEM1	MT230534.1	*Myoviridae*	54982	unpublished
vB_PagM_AAM37	MK798143.1	*Myoviridae*	49990	unpublished
vB_PagM_PSKM	MK798144.1	*Myoviridae*	49935	unpublished
vB_PagM_AAM22	MK798142.1	*Myoviridae*	49744	unpublished
**vB_PagS_AAS21**	**MK770119.1**	***Siphoviridae***	**116649**	[this study]
vB_PagS_Vid5	MG948468.1	*Siphoviridae*	61437	[6]
vB_PagS_AAS23	MK095606.1	*Siphoviridae*	51170	unpublished
vB_PagS_MED16	MK095605.1	*Siphoviridae*	46103	unpublished
LIMElight	FR687252.1	*Podoviridae*	44546	[5]
LIMEzero	FR751545.1	*Podoviridae*	43032	[5]
vB_PagP-SK1	MN450150.1	*Podoviridae*	39938	[8]

Supplementary Table S2. Bacterial strains used in this study to determine the host range of phage AAS21.

**Strain**	**Relevant characteristics**	**Source or reference**
Acinetobacter baumannii #46		Prof. E. Sužiedelienė
Citrobacter freundii		Prof. E. Sužiedelienė
Erwinia billingiae, Mergaert et al. 1999	DSM 17872, Type strain	DSMZ
Erwinia carotovora 8982		Prof. E. Sužiedelienė
Erwinia carotovora 961–63		Prof. E. Sužiedelienė
Erwinia piriflorinigrans, López et al. 2011	DSM 26166, Type strain	DSMZ
Escherichia coli B40	*sup*D	Dr. L. W. Black
Escherichia coli B^E	*sup*^{0}	Dr. L. W. Black
Escherichia coli BL21	F^- *dcm ompT hsdS* (rB^-mB^-) *gal*	Novagen
Escherichia coli DH10β	F^- *endA1 recA1 galE15 galK16 nupG rpsL* Δ*lacX74* Φ80*lacZ*ΔM15 *araD139* Δ(*ara,leu*)7697 *mcrA* Δ(*mrr-hsdRMS-mcrBC*) λ^-	Invitrogen
Escherichia coli MG1655	F^- lambda$^-$ *ilvG*$^-$ *rfb*-50 *rph*-1	Prof. E. Sužiedelienė
Escherichia coli MH1	*araD139* Δ*lacX74 galU galK hsr hsm rpsL*	Dr. K. N. Kreuzer
Klebsiella sp. KV-3	Veterinary isolate, Amp^r, Str^r, Tet^r, Kan^s, Gm^s, Nc^s,$Cl^{r/s}$	[10]
Pantoea agglomerans (Beijerinck 1888), Gavini et al. 1989	DSM 3493, Type strain	DSMZ
Pantoea agglomerans ARC	environmental isolate	[6]
***Pantoea agglomerans* AUR**	environmental isolate	[6]
***Pantoea agglomerans* BSL**	environmental isolate	[6]
Pantoea agglomerans DDM	environmental isolate	[6]
Pantoea agglomerans MMG	environmental isolate	[6]
***Pantoea agglomerans* SER**	environmental isolate	[6]
Pantoea conspicua, Brady et al. 2010	DSM 24241, Type strain	DSMZ
Pseudomonas aeruginosa PAO1		Prof. E. Sužiedelienė
Salmonella enterica ser. Typhimurium 292		Prof. E. Sužiedelienė

AAS21-sensitive strains are marked in bold.

Supplementary Table S3. AAS21 ORFs with homologues in other viruses or cellular organisms.

**AAS21 ORF (position)**	**Predicted function (protein length aa)**	**Significant match (protein length aa)**	**Identity aa %/ similarity aa% (length of the overlapping segment)**	**E-value**
ORF001 (1..480)	terminase small subunit (159)	QFR57577.1 hypothetical protein JIPhKp127_0169 *Klebsiella* phage JIPh_Kp127 (157)	62/71 (156)	1e-61
ORF002 (480..1796)	terminase large subunit (438)	QEG11189.1 terminase large subunit *Klebsiella* phage KPN4 (438)	85/91 (438)	0.0
ORF003 (1972..2415)	hypothetical protein (147)	YP_004306623.1 hypothetical protein SPC35_0140 *Salmonella* virus SPC35 (145)	81/84 (145)	2e-82
ORF004 (2405..3622)	portal protein (405)	YP_007237120.1 portal protein *Yersinia* phage phiR201 (408)	70/86 (405)	0.0
ORF005 (3625..4101)	structural protein containing Ig-like domain (158)	YP_007237119.1 phage neck whiskers *Yersinia* phage phiR201 (158)	56/68 (160)	1e-47
ORF006 (4103..4744)	prohead protease (213)	QEG07474.1 capsid protein *Salmonella* phage SE3 (210)	67/79 (211)	5e-100
ORF007 (4744..6132)	major capsid protein (462)	VCU43541.1 major head protein precursor *Escherichia* virus vB_Eco_mar004NP2 (458)	68/81 (457)	0.0
ORF008 (6196..6705)	putative head-tail connector (169)	YP_004306618.1 hypothetical protein SPC35_0135 *Salmonella* virus SPC35 (170)	40/58 (172)	9e-34
ORF009 (6707..7480)	putative tail completion protein (257)	VUF55653.1 tail completion protein *Escherichia* virus T5 (255)	64/76 (259)	2e-107
ORF010 (7480..7959)	tail terminator protein (159)	AWN08781.1 tail terminator protein *Escherichia* phage Gostya9 (161)	62/80 (157)	2e-71
ORF011 (7982..9379)	major tail protein (465)	YP_007237113.1 phage major tail protein *Yersinia* phage phiR201 (466)	65/77 (453)	0.0
ORF012 (9388..1028)	putative minor tail protein (297)	AXC41331.1 minor tail protein *Salmonella* phage S126 (299)	48/67 (297)	5e-103
ORF013 (10281..1068)	putative tape measure chaperone (133)	YP_009283414.1 hypothetical protein NR01_0073 *Salmonella* phage NR01 (134)	47/69 (133)	1e-38
ORF014 (10657..11118)	putative tape measure chaperone (153)	AXY85088.1 tape measure chaperone *Salmonella* phage Sw2 (277)	50/67 (146)	4e-44
ORF015 (11199..14900)	tape measure protein (1233)	QEQ93451.1 tail length tape-measure protein *Salmonella* phage 2-3 (1235)	51/67 (1255)	0.0
ORF016 (15018..15632)	distal tail protein (204)	AWN08775.1 distal tail protein *Escherichia* phage Gostya9 (204)	65/81 (203)	5e-96
ORF017 (15629..18481)	putative tail protein (950)	AXC41489.1 hypothetical protein *Salmonella* phage S130 (949)	68/83 (951)	0.0
ORF018 (18466..20541)	putative tail protein (691)	YP_009202112.1 hypothetical protein SLUR09_00066 *Escherichia* phage slur09 (694)	65/79 (696)	0.0
ORF019 (20546..20971)	putative tail protein (141)	AXC41338.1 hypothetical protein *Salmonella* phage S126 (140)	49/62 (140)	6e-41

ORF020 (20971..23811)	tail fiber protein (946)	ASV44964.1 hypothetical protein MezzoGao_18 *Klebsiella* phage MezzoGao (973)	40/51 (887)	2e-151
ORF21 (23846..25801)	tail fiber protein (651)	AVO22985.1 minor tail protein *Erwinia* phage vB_EamM-Bue1 (872)	55/67 (547)	3e-179
ORF022 (25842..26297)	deoxyUTP pyrophosphatase (151)	AXY85079.1 deoxyUTP pyrophosphatase *Salmonella* phage Sw2 (148)	58/74 (147)	2e-57
ORF023 (26284..2716)	exodeoxy ribonuclease (292)	QGH45232.1 ribonuclease bacteriophage Eos (290)	60/74 (294)	2e-126
ORF024 (27159..27644)	putative holliday junction resolvase (161)	QFR57721.1 D14 protein *Serratia* phage Slocum (160)	68/79 (159)	6e-78
ORF025 (27644..29497)	recombination-related endonuclease (617)	ASD50273.1 exonuclease subunit 2 *Shigella* phage SSP1 (612)	56/75 (617)	0.0
ORF026 (29478..30461)	recombination-related endonuclease (327)	QGH45235.1 DNA repair exonuclease bacteriophage Eos (328)	64/79 (325)	5e-154
ORF027 (30504..31277)	putative ssDNA binding protein (257)	YP_009597487.1 hypothetical protein *Klebsiella* phage vB_Kpn_IME260 (257)	55/69 (256)	3e-94
ORF028 (31270..31665)	hypothetical protein (131)	QEG11214.1 hypothetical protein KPN4_28 *Klebsiella* phage KPN4 (117)	54/76 (116)	3e-43
ORF029 (31753..33105)	ATP-dependent DNA helicase (450)	AXC43079.1 DNA helicase *Salmonella* phage S124 (450)	62/75 (449)	0.0
ORF030 (33102..33599)	hypothetical protein (165)	YP_009599156.1 hypothetical protein PR1_124 *Providencia* phage vB_PreS_PR1 (164)	52/73 (162)	8e-54
ORF031 (33589..36159)	DNA polymerase I (856)	YP_009146069.1 DNA polymerase *Salmonella* virus Stitch (855)	76/86 (856)	0.0
ORF032 (36220..37110)	DNA primase (296)	ASM62927.1 putative DNA replication primase *Escherichia* phage OSYSP (296)	69/84 (294)	2e-157
ORF033 (37110..38591)	replicative DNA helicase (493)	YP_004306593.1 putative replicative DNA helicase *Salmonella* virus SPC35 (507)	61/80 (492)	0.0
ORF034 (38661..39419)	putative transcription factor (252)	YP_009621051.1 late gene transcription factor *Klebsiella* phage Sugarland (252)	65/79 (251)	2e-112
ORF035 (39426..40193)	NAD-dependent DNA ligase, subunit B (255)	YP_009597480.1 NDP-dependent DNA ligase subunit B *Klebsiella* phage vB_Kpn_IME260 (249)	62/76 (247)	2e-109
ORF037 (40661..41644)	NAD-dependent DNA ligase, subunit A (327)	AXC43086.1 NAD-dependent DNA ligase subunit A *Salmonella* phage S124 (324)	70/81 (329)	2e-168
ORF038 (41620..42180)	putative DNA processing protein (186)	ATS94074.1 hypothetical protein P13BB106kb_p090 *Pectobacterium* phage DU_PP_V (187)	57/74 (179)	7e-69
ORF039 (42167..42463)	putative nucleoside triphosphate pyrophosphohydrolase (98)	YP_006906361.1 hypothetical protein My1_109 *Pectobacterium* phage My1 (106)	41/58 (85)	2e-13
ORF040 (42463..42708)	hypothetical protein (81)	ATI18466.1 hypothetical protein *Salmonella* phage SP1a (90)	40/53 (90)	7e-11
ORF042 (43001..43309)	putative transcriptional coactivator (102)	YP_004306588.1 hypothetical protein SPC35_0105 *Salmonella* virus SPC35 (102)	61/82 (96)	8e-40
ORF043 (43287..43625)	hypothetical protein (112)	YP_006906359.1 hypothetical protein My1_107 *Pectobacterium* phage My1 (104)	65/75 (104)	3e-43

ORF044 (43635..44030)	hypothetical protein (131)	YP_006941.1 D3 protein *Escherichia* virus T5 (136)	61/78 (130)	3e-51
ORF045 (44130..44888)	hypothetical protein (252)	ATS94070.1 D2 protein *Pectobacterium* phage DU_PP_V (232)	56/72 (237)	8e-86
ORF046 (44902..45132)	hypothetical protein (76)	ARQ96334.1 hypothetical protein *Salmonella* phage Stp1 (86)	39/62 (72)	3e-10
ORF047 (45122..47908)	putative ATP-dependent RNA helicase (928)	AKO61520.1 helicase *Escherichia* phage APCEc03 (928)	75/86 (927)	0.0
ORF051 (49009..49758)	Sir2-like protein (249)	YP_006906350.1 Sir2-like protein *Pectobacterium* phage My1 (251)	72/81 (248)	4e-133
ORF052 (49751..50107)	hypothetical protein (118)	ARB06989.1 hypothetical protein *Escherichia* phage phiLLS (119)	44/65 (75)	2e-12
ORF053 (50109..50618)	hypothetical protein (169)	AYN56010.1 hypothetical protein STG2_46 *Salmonella* phage STG2 (168)	44/61 (157)	1e-37
ORF054 (50888..51664)	PhoH-like protein (258)	YP_009194732.1 phosphate starvation inducible protein *Salmonella* phage Shivani (250)	70/82 (258)	1e-131
ORF055 (51664..51906)	hypothetical protein (80)	VUF55420.1 phage protein *Escherichia* virus T5 (80)	51/62 (59)	4e-14
ORF056 (52033..54363)	ribonucleoside-diphosphate reductase, alpha subunit (776)	AXC41827.1 ribonucleotide reductase of class Ia (aerobic), alpha subunit *Salmonella* phage S132 (777)	62/78 (779)	0.0
ORF057 (54465..55613)	ribonucleoside-diphosphate reductase, beta subunit (382)	QEG11241.1 ribonucleoside-diphosphate reductase subunit beta *Klebsiella* phage KPN4 (385)	52/67 (387)	9e-130
ORF058 (55613..56179)	dihydrofolate reductase (188)	ARM69772.1 putative dihydrofolate reductase *Salmonella* phage BSP22A (176)	39/52 (189)	7e-32
ORF059 (56166..57017)	thymidylate synthase (283)	QFR57691.1 thymidylate synthase *Serratia* phage Slocum (277)	67/80 (284)	2e-139
ORF060 (57106..57678)	protease (190)	AXC43114.1 proteosome subunit *Salmonella* phage S124 (192)	52/68 (190)	7e-65
ORF061 (57671..58144)	ribonuclease H (157)	QFR57689.1 RNaseH ribonuclease *Serratia* phage Slocum (162)	56/68 (160)	2e-56
ORF064 (58745..59032)	hypothetical protein (95)	YP_007237059.1 hypothetical protein BN79_099 *Yersinia* phage phiR201 (92)	72/81 (92)	1e-41
ORF065 (59128..59631)	hypothetical protein (167)	YP_001837022.1 hypothetical protein AGC_0099 *Escherichia* virus EPS7 (171)	49/64 (154)	1e-35
ORF066 (59670..59927)	hypothetical protein (85)	ASD50315.1 hypothetical protein SSP1_144 *Shigella* phage SSP1 (86)	42/58 (78)	4e-16
ORF067 (59924..60136)	hypothetical protein (70)	VCU43749.1 hypothetical protein MAR003J3_00027 *Escherichia* virus vB_Eco_mar003J3 (70)	62/83 (66)	5e-25
ORF068 (60130..60864)	putative metallopeptidase (244)	QBX06947.1 putative metallopeptidase *Klebsiella* phage Spivey (304)	36/53 (254)	2e-43
ORF069 (60932..61117)	hypothetical protein (61)	AWN08725.1 hypothetical protein T59_00076c *Escherichia* phage Gostya9 (60)	51/65 (61)	6e-10
ORF070 (61163..61804)	hypothetical protein (213)	QFR57681.1 hypothetical protein CPT_Slocum_124 *Serratia* phage Slocum (213)	51/67 (208)	5e-60
ORF071 (62233..62556)	hypothetical protein (107)	QBQ81360.1 hypothetical protein HASG4_00107 *Escherichia* phage vB_EcoS_HASG4 (105)	70/82 (103)	1e-45

ORF072 (62568..63002)	cell wall hydrolase (144)	AXC43124.1 cell wall hydrolyse *Salmonella* phage S124 (149)	59/70 (131)	3e-49
ORF073 (63002..63226)	hypothetical protein (74)	QFR57679.1 hypothetical protein CPT_Slocum_122 *Serratia* phage Slocum (72)	43/60 (60)	6e-10
ORF075 (63460..63885)	YqeY-like protein (141)	QDK00026.1 hypothetical protein HEDJPLGI_00135 *Escherichia* phage vB_EcoS-26175I (144)	35/53 (141)	1e-16
ORF079 (64843..65304)	hypothetical protein (153)	YP_009624036.1 hypothetical protein *Yersinia* phage fHe-Yen9-04 (154)	25/50 (151)	3e-12
ORF080 (65402..66481)	hypothetical protein (359)	ATS94045.1 hypothetical protein P13BB106kb_p061 *Pectobacterium* phage DU_PP_V (363)	57/62 (374)	1e-151
ORF081 (66468..67049)	hypothetical protein (193)	YP_009599113.1 hypothetical protein PR1_82 *Providencia* phage vB_PreS_PR1 (189)	63/75 (184)	4e-81
ORF086 (68770..68928)	hypothetical protein (52)	ATI99460.1 hypothetical protein *Salmonella* phage SP01 (52)	67/75 (52)	9e-25
ORF088 (69193..69270)	hypothetical protein (25)	QEQ94943.1 putative terminase large subunit *Erwinia* phage pEp_SNUABM_01 (30)	83/83 (12)	8e-04
ORF089 (69356..69556)	hypothetical protein (66)	ATS94041.1 hypothetical protein P13BB106kb_p057 *Pectobacterium* phage DU_PP_V (64)	63/65 (38)	3e-10
ORF090 (69634..69855)	hypothetical protein (73)	YP_007237039.1 g060 *Yersinia* phage phiR201 (74)	77/86 (73)	2e-35
ORF091 (69871..70041)	hypothetical protein (56)	AXC40642.1 hypothetical protein *Salmonella* phage S116 (63)	67/68 (45)	1e-20
ORF095 (70800..70964)	hypothetical protein (54)	AXN57755.1 hypothetical protein *Acinetobacter* phage ABPH49 (56)	45/51 (64)	3e-10
ORF096 (71071..71415)	hypothetical protein (114)	YP_007237044.1 hypothetical protein BN79_081 *Yersinia* phage phiR201 (117)	54/74 (112)	1e-37
ORF097 (71412..71681)	hypothetical protein (89)	EAT0097302.1 hypothetical protein *Salmonella enterica* (82)	61/63 (80)	4e-31
ORF099 (72650..72811)	hypothetical protein (53)	ATS94039.1 hypothetical protein P13BB106kb_p055 *Pectobacterium* phage DU_PP_V (67)	39/57 (49)	7e-05
ORF101 (73208..73663)	endonuclease V (151)	AWD92089.1 endonuclease V N-glycosylase UV repair enzyme Enterobacteria phage vB_EcoM_IME341 (138)	56/62 (140)	1e-48
ORF104 (74076..74456)	HNH endonuclease (126)	YP_009283342.1 putative HNH nuclease domain-containing protein *Salmonella* phage NR01 (128)	74/75 (124)	5e-84
ORF105 (74540..74689)	hypothetical protein (49)	YP_009599110.1 hypothetical protein PR1_79 *Providencia* phage vB_PreS_PR1 (49)	60/68 (35)	7e-10
ORF111 (75342..75818)	hypothetical protein (158)	EBX7861945.1 hypothetical protein *Salmonella enterica* subsp. enterica serovar Bareilly (72)	78/83 (59)	7e-34
ORF112 (76187..76384)	hypothetical protein (65)	YP_009646877.1 hypothetical protein *Escherichia* virus BF23 (73)	64/73 (61)	1e-27
ORF115 (77152..77229)	hypothetical protein (25)	QCQ65572.1 hypothetical protein Sepoy_096 *Salmonella* phage Sepoy (57)	88/94 (17)	1e-08

ORF117 (77717..78184)	HNH endonuclease (155)	ARQ95798.1 HNH endonuclease protein *Staphylococcus* phage qdsa001 (238)	41/47 (158)	6e-22
ORF119 (78474..78707)	hypothetical protein (77)	YP_009145995.1 hypothetical protein CPT_Stitch54 *Salmonella* virus Stitch (99)	40/43 (87)	4e-09
ORF120 (78783..78980)	hypothetical protein (65)	YP_009320781.1 hypothetical protein *Salmonella* phage 100268_sal2 (61)	48/57 (66)	2e-12
ORF121 (79039..79401)	hypothetical protein (120)	YP_007237025.1 hypothetical protein BN79_046 *Yersinia* phage phiR201 (117)	47/51 (131)	2e-26
ORF122 (79401..79628)	hypothetical protein (75)	QBZ72709.1 hypothetical protein SEA_GODONK_90 *Gordonia* phage GodonK (67)	53/62 (51)	1e-12
ORF123 (79691..79981)	hypothetical protein (96)	AYN56069.1 hypothetical protein STG2_105 *Salmonella* phage STG2 (91)	48/55 (92)	2e-21
ORF126 (80231..80791)	hypothetical protein (186)	YP_006906297.1 hypothetical protein My1_045 *Pectobacterium* phage My1 (109)	51/56 (83)	4e-12
ORF127 (80860..81174)	hypothetical protein (104)	YP_006906298.1 hypothetical protein My1_046 *Pectobacterium* phage My1 (114)	53/60 (89)	4e-22
ORF128 (81174..81440)	hypothetical protein (88)	WP_110877013.1 SOS-response repressor and protease LexA *Franconibacter helveticus* (161)	40/46 (100)	3e-06
ORF129 (81552..81827)	hypothetical protein (91)	AYN56073.1 putative membrane protein *Salmonella* phage STG2 (94)	53/60 (38)	1e-05
ORF130 (81940..82629)	hypothetical protein (229)	AVQ09939.1 hypothetical protein *Salmonella* phage vB_SenS_PHB06 (226)	38/43 (286)	4e-50
ORF132 (83036..83374)	hypothetical protein (112)	YP_006872.1 hypothetical protein T5.044 *Escherichia* virus T5 (117)	47/54 (116)	2e-24
ORF133 (83371..84093)	deoxynucleoside-5'-monophosphate kinase (240)	QEI24727.1 deoxynucleoside-5'-monophosphate kinase *Salmonella* phage SE19 (250)	40/46 (251)	2e-36
ORF134 (84102..84716)	ATP-dependent Clp protease (204)	QFR57640.1 ATP-dependent Clp protease *Serratia* phage Slocum (223)	52/57 (181)	2e-54
ORF135 (84868..85530)	holin (220)	QBQ81141.1 putative holin *Escherichia* phage vB_EcoS_HdH2 (218)	65/ 73 (221)	1e-127
ORF136 (85527..86024)	lysozyme (165)	YP_006987338.1 baseplate hub subunit and tail lysozyme *Cronobacter* phage vB_CsaM_GAP32 (162)	50/57 (161)	2e-41
ORF138 (86588..86974)	hypothetical protein (128)	QBQ80654.1 hypothetical protein VAH1_00026 *Escherichia* phage vB_EcoS_VAH1 (134)	48/53 (141)	1e-32
ORF139 (86971..87258)	thioredoxin (95)	QCG76519.1 thioredoxin *Klebsiella* phage vB_KpnS_FZ41 (94)	46/52 (71)	8e-10
ORF142 (87917..88291)	hypothetical protein (124)	YP_004306518.1 hypothetical protein SPC35_0035 *Salmonella* virus SPC35 (125)	42/45 (93)	4e-11
ORF143 (88291..89118)	serine/threonine protein phosphatase (275)	QEG07588.1 serine/threonine protein phosphatase *Salmonella* phage SE3 (287)	38/45 (282)	1e-39
ORF145 (89388..89633)	hypothetical protein (81)	YP_009324977.1 hypothetical protein GA2A_41 *Escherichia* phage vB_EcoP_GA2A (75)	51/60 (73)	2e-13
ORF146	hypothetical protein (94)	YP_006906283.1 putative protein 2C	50/63 (74)	5e-14

(89695..89979)		*Pectobacterium* phage My1 (85)		
ORF151 (91002..91439)	hypothetical protein (145)	ARB06941.1 hypothetical protein lls_113 *Escherichia* phage phiLLS (153)	50/57 (143)	7e-40
ORF153 (91892..92305)	hypothetical protein (137)	EBX7861978.1 hypothetical protein *Salmonella enterica* subsp. enterica serovar Bareilly (99)	46/56 (89)	2e-07
ORF154 (92305..95433)	tail fiber protein–EPS-depolymerase (1042)	QEQ94917.1 putative EPS-depolymerase *Erwinia* phage pEp_SNUABM_01 (859)	52/56 (836)	0.0
ORF160 (96403..96819)	hypothetical protein (138)	QFG07697.1 hypothetical protein *Salmonella* phage vB_SenS_SB13 (172)	66/71 (134)	2e-66
ORF161 (96803..97114)	hypothetical protein (103)	YP_009604641.1 hypothetical protein vBPaeMG1_014 *Pseudomonas* phage vB_PaeM_G1 (377)	53/56 (51)	3e-09
ORF166 (98273..98611)	putative peptidyl-tRNA hydrolase (112)	AMR58012.1 hypothetical protein vB_PsyM_KIL5_0121 *Pseudomonas* phage vB_PsyM_KIL5 (114)	68/81 (110)	4e-51
ORF167 (98586..98753)	hypothetical protein (55)	ASV43975.1 hypothetical protein *Pseudoalteromonas* phage KB12-38 (51)	47/70 (51)	6e-12
ORF176 (100520..100942)	hypothetical protein (140)	AZS06388.1 hypothetical protein AAS23_gp75 *Pantoea* phage vB_PagS_AAS23 (151)	37/52 (115)	4e-13
ORF180 (102314..103030)	hypothetical protein (238)	QEA11093.1 hypothetical protein Th1_012 *Salmonella* phage Th1 (251)	34/41 (272)	3e-16
ORF192 (104858..105088)	hypothetical protein (76)	ARM69675.1 hypothetical protein BSP22A_0012 *Salmonella* phage BSP22A (75)	60/64 (67)	9e-22
ORF197 (105952..106668)	hypothetical protein (238)	ASU01463.1 hypothetical protein P24_0010 bacteriophage T5-like chee24 (164)	44/54 (66)	5e-07
ORF198 (106733..107221)	hypothetical protein (162)	QFR57442.1 hypothetical protein JIPhKp127_0012 *Klebsiella* phage JIPh_Kp127 (164)	40/45 (107)	7e-10
ORF199 (107289..108239)	hypothetical protein (316)	ARQ96276.1 hypothetical protein *Salmonella* phage Stp1 (325)	53/57 (146)	3e-43
ORF202 (109833..110072)	hypothetical protein (79)	QBQ81105.1 hypothetical protein HdH2rev_00007 *Escherichia* phage vB_EcoS_HdH2 (83)	74/82 (78)	4e-55
ORF204 (110236..110661)	A2 protein (141)	QFG07522.1 A2 protein *Salmonella* phage vB_SenS_SB10 (138)	55/57 (138)	2e-35
ORF206 (111038..112699)	A1 protein (553)	ATW62529.1 DNA transfer protein *Salmonella* phage SP3 (554)	66/71 (570)	0.0
ORF207 (112792..113214)	hypothetical protein (140)	QBX06863.1 hypothetical protein CPT_Spivey_005 *Klebsiella* phage Spivey (150)	54/63 (46)	7e-08
ORF208 (113290..113985)	deoxynucleoside-5'-monophosphatase (231)	VCU43828.1 deoxynucleoside-5'-monophosphatase *Escherichia* virus vB_Eco_mar003J3 (244)	57/61 (236)	4e-91
ORF212 (114464..114586)	hypothetical protein (40)	ATW62678.1 hypothetical protein *Salmonella* phage SP3 (45)	74/77 (31)	2e-15
ORF213 (114691..116640)	receptor binding protein (649)	P_009202094.1 hypothetical protein SLUR09_00048 *Escherichia* phage slur09 (639)	35/38 (415)	5e-39

Supplementary Table S4. AAS21 virion proteins identified by MS.

Gene product	Predicted function	MW (kDa)	Sequence coverage (%)	Number of unique peptides
gp004	portal protein	45.030	42.7	22
gp005	structural protein containing Ig-like domain	16.293	42.4	10
gp006	prohead protease	23.578	35.2	9
gp007	major capsid protein	51.167	30.9	15
gp011	major tail protein	50.052	22.8	9
gp015	tape measure protein	131.561	37.7	43
gp018	putative tail protein	74.897	15.8	9
gp019	putative tail protein	15.308	28.4	4
gp020	tail fiber protein	100.300	26.4	19
gp021	tail fiber protein	67.983	26.1	11
gp070	hypothetical protein	23.105	10.3	2
gp080	hypothetical protein	40.932	27.6	8
gp081	hypothetical protein	21.700	14.5	3
gp135	holin	24.973	27.3	6
gp147*	hypothetical protein	11.037	16.3	2
gp153	hypothetical protein	15.268	70.1	9
gp154	tail fiber protein–EPS-depolymerase	110.701	60.7	121
gp172*	hypothetical protein	8.770	42.1	3
gp177*	hypothetical protein	11.413	31.9	3

* – AAS21 specific ORFs that encode unique proteins having no reliable identity to database entries.

Supplementary Table S5. AAS21 tRNAs identified with tRNAscan-SE and ARAGORN.

tRNA	tRNA Begin	tRNA End	tRNA Type	Anticodon	Infernal Score
1	64096 (64095)*	64166 (64167)*	Ser	CGA (AGA)*	31.5
2	65311	65385	Arg	TCT	65.3
3	67165	67252	Ser	GCT	62.3
4	67257	67334	Met	CAT	42.2
5	67342	67427	Leu	TAA	54.9
6	67701	67786	Leu	TAG	55.9
7	68492	68578	Tyr	GTA	57.3
8	68677	68752	Glu	TTC	61.8
9	68930	69006	Phe	GAA	64.7
10	69274	69344	Trp	CCA	30.8
11	70230	70306	Cys	GCA	33.8
12	70312 (70314)*	70389	Asn	GTT	51.7
13	70481	70563	Leu	TAG	22.3
14	70972	71049	Pro	TGG	75.0
15	71672	71750 (71749)*	Met	CAT	38.3
16	71968	72044	Lys	TTT	66.8
17	72051	72126	Val	TAC	63.5
18	72289	72364	Asp	GTC	62.5
19	72368	72440	Undet	NNN	41.7
20	74447	74522	Ala	TGC	51.0
21	74698	74791	Ser	TGA	42.6
22	75248	75325	His	GTG	50.8
23	74698	74791	Ser	TGA	42.6
24	75828	75905	Arg	ACG	68.6
25	76013 (76095)*	76088	Gln	CTG	61.0
26	76096	76169	Gln	TTG	53.3
27	76803	76877	Gly	TCC	55.2
28	77266	77339	Thr	TGT	55.8
29	77637	77712	Ile	GAT	59.8

* – tRNA start/stop positions and anticodon identified with ARAGORN.

Supplementary Table S6. Top matches for BLAST-based alignments of the whole genome sequences of AAS21 and its closest relatives generated using PASC.

The overall nucleotide sequence identity (%)	Reference Sequence	Bacteriophage	Genus
41.16	gi|388570360|ref|NC_017969.1	*Escherichia* virus AKFV33	Tequintavirus
40.85	gi|640884271|ref|NC_024139.1	*Escherichia* phage vB_EcoS_FFH_1	Tequintavirus
40.81	gi|182682799|ref|NC_010583.1	*Escherichia* virus EPS7	Tequintavirus
39.99	gi|971820626|ref|NC_028840.1	*Escherichia* phage slur09	Tequintavirus
39.91	gi|849253165|ref|NC_027356.1	*Escherichia* virus DT57C	Tequintavirus
39.61	gi|847897049|ref|NC_027297.1	*Salmonella* virus Stitch	Tequintavirus
39.46	gi|1102616974|ref|NC_031902.1	*Salmonella* phage 100268_sal2	Tequintavirus
39.28	gi|1006160387|ref|NC_019919.2	*Yersinia* phage phiR201	Tequintavirus
39.23	gi|326632894|ref|NC_015269.1	*Salmonella* virus SPC35	Tequintavirus
38.94	gi|1070097795|ref|NC_031022.1	*Shigella* phage SHSML-45	Tequintavirus
38.87	gi|1070099636|ref|NC_031042.1	*Salmonella* phage NR01	Tequintavirus
38.74	gi|1666231604|ref|NC_042307.1	*Escherichia* virus H8	Tequintavirus
38.1	gi|46401737|ref|NC_005859.1	*Escherichia* virus T5	Tequintavirus
37.72	gi|1631943254|ref|NC_042093.1	*Klebsiella* phage Sugarland	Sugarlandvirus
36.65	gi|971740341|ref|NC_028754.1	*Salmonella* phage Shivani	Tequintavirus

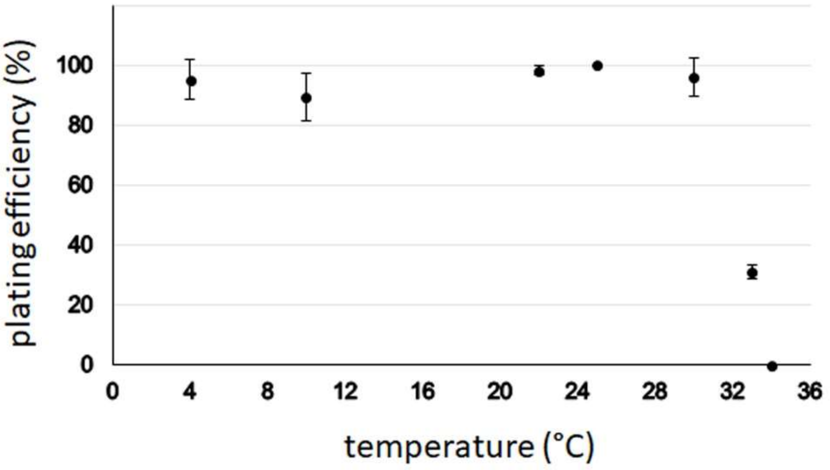

Supplementary Figure S1. The effect of temperature on the efficiency of plating of phage AAS21 on the culture of *P. agglomerans* strain AUR. Each point represents the mean of three individual experiments with error bars showing the variation between each experiment.

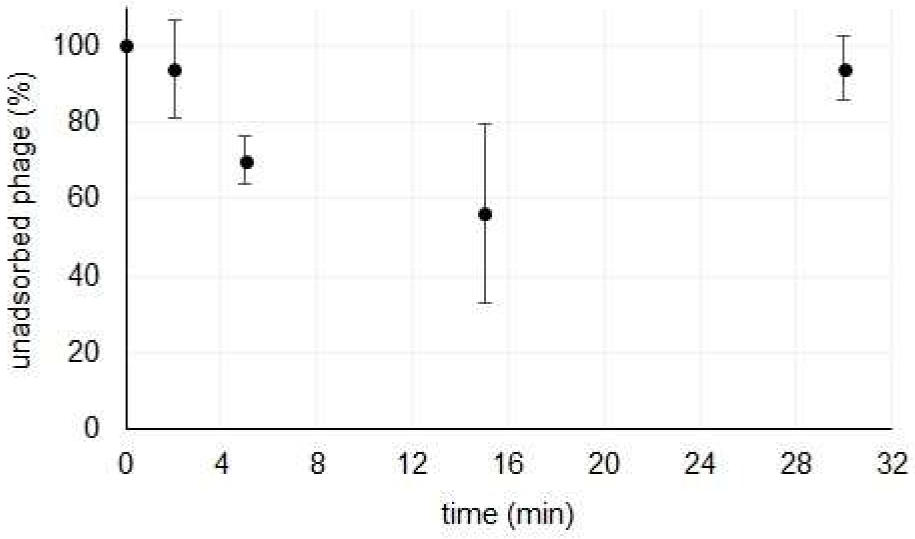

Supplementary Figure S2. Adsorption assay of phage AAS21. Phage (MOI=10) was added to the mid-log phase culture of *P. agglomerans* strain AUR. The experiments were performed in triplicate with error bars showing the variation between each experiment.

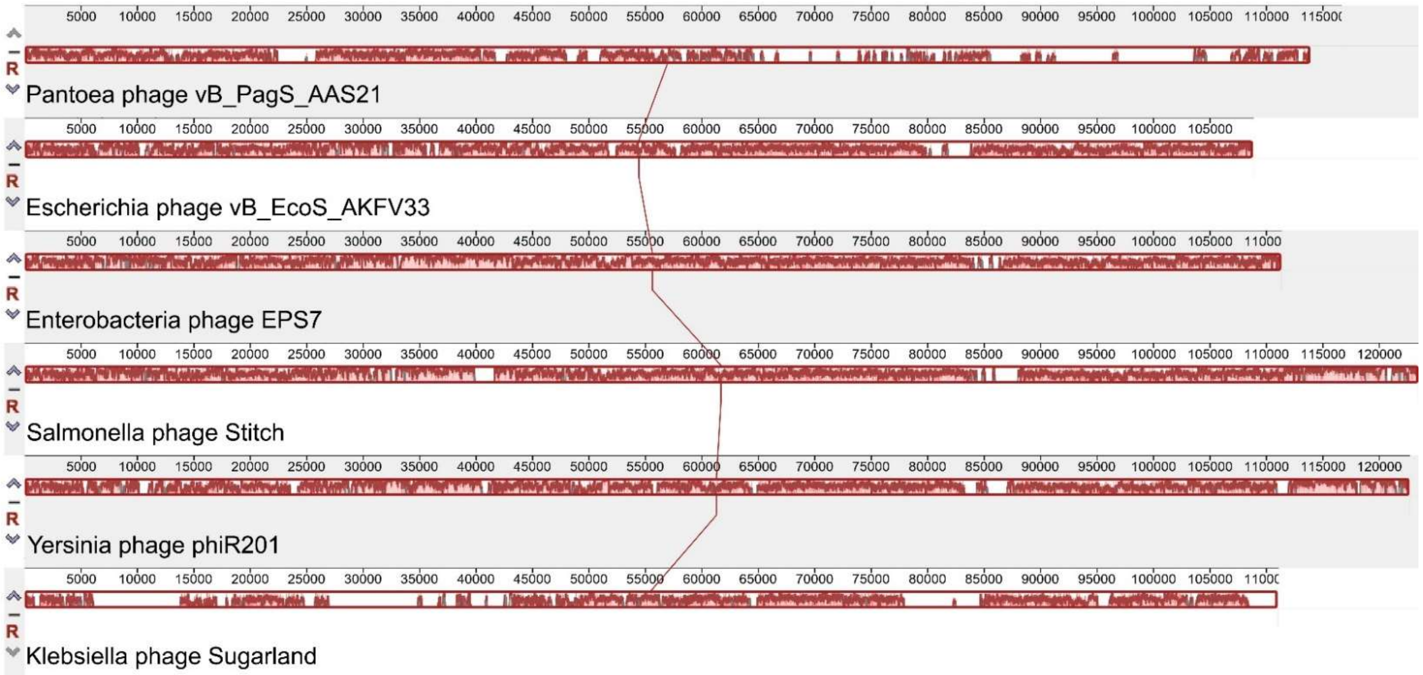

Supplementary Figure S3. Progressive Mauve whole-genome alignment generated using Geneious Prime 2019. Red blocks represent aligned regions, and similarity is indicated by the height of the bars.
